# Building skill‐sets, confidence, and interest for diverse scientific careers in the biological and biomedical sciences

**DOI:** 10.1096/fba.2021-00087

**Published:** 2021-09-15

**Authors:** Jennifer Claydon, Katherine Farley‐Barnes, Susan Baserga

**Affiliations:** ^1^ Training Program Assessment, Biological & Biomedical Sciences & Poorvu Center for Teaching and Learning Yale University New Haven Connecticut USA; ^2^ Program Development for the Cellular, Molecular, and Quantitative Biology Training Grant Molecular Biophysics & Biochemistry Yale University New Haven Connecticut USA; ^3^ William H. Fleming MD Professor of Molecular Biophysics & Biochemistry, Genetics, & Therapeutic Radiology Yale University New Haven Connecticut USA

**Keywords:** career development, evaluation, graduate training, job skills, STEM

## Abstract

Many biological science PhD graduates are increasingly pursuing careers outside of academia. Subsequently, PhD training programs are increasing their efforts to broaden their awareness of diverse career opportunities, with a firm knowledge of the skills necessary for success. At Yale University, for two semesters we have offered a new course for graduate students in the biological sciences titled “Skills Development for Diverse Scientific Careers” (BBS 550b). This course addressed career‐related topics not covered in any curriculum at Yale such as how to run clinical trials, the business side of biotech, how to convert CVs into resumes, and resilience for early career scientists. We sought to better equip students to think broadly about their career options by exposing them to non‐academic biomedical career avenues. Furthermore, the course fulfilled a gap in current curricular offerings to prepare students for multiple science career trajectories. Results on a pre‐post course survey demonstrated increases in students’ interest for, knowledge of, and confidence in securing a position in multiple nontraditional career sectors. Intentional course design can provide an adequate foundation to broaden awareness of myriad career options available to bioscientists. Broadening student knowledge and interest levels will contribute substantially to developing a robust scientific workforce.

## 1 INTRODUCTION

Since the inception of doctoral education in the 1800s in America, the number of individuals pursuing graduate degrees has continued to increase. In 2019, 55,703 doctorates were awarded in Science, Technology, Engineering, and Math (STEM) fields alone, with 11,290 of these specific to Biological and Biomedical Sciences and Health Sciences, according to the National Science Foundation (NSF) Survey of Earned Doctorates.^1^ However, the biomedical workforce is changing with many students exploring career options outside of the more traditional academic options. A reported 73% of available instructional positions in 2016 were off the tenure‐track, increasing the competitiveness and the depth of training required for obtaining a professorship.^2^, ^3^, ^4^ In addition, a majority of recent doctoral graduates have been found pursuing positions outside of academia,^5^ and those graduates who pursue careers outside of academia have reported that their training did not fully prepare them for those positions.^6^, ^7^ With the continuation of the COVID 19 pandemic, many institutions and companies have initiated hiring freezes, reducing available positions for graduates and possibly forcing current students to reexamine their career goals. In response to the current state of bioscience career prospects and increased student desire in diverse careers, the calls for modernized or revolutionized graduate education have been more frequent and given more credence.^8^

Due to nationally shifting economic needs and the realities of the job market, many educators have advocated for updated doctoral education models.^9^, ^10^ One promising new avenue for significant curricular reform seeks to increase focus on skills‐based training.^11^, ^12^, ^13^ Advocates of this approach suggest that skills‐based instruction across a spectrum of aptitudes will better prepare graduates for multiple career opportunities.^14^, ^15^, ^16^, ^17^, ^18^, ^19^, ^20^

Calls for graduate curricular reform have been echoed at the national level in the United States.^21^, ^22^ The new NIGMS T32 training grant proposal structure requires articulated initiatives for how the training program will enhance the career development of trainees.^23^ In 2013 and 2014, the NIH awarded funding to 17 institutions through the Broadening Experiences in Scientific Training (BEST) initiative.^24^, ^25^ Although the outcomes from this program are preliminary, the data suggest that BEST program participants gain exposure to a diversity of careers as well as the confidence to achieve their career goals.^25^ Smaller scale initiatives have also effectively prepared students for a changing economic environment.^26^, ^27^ These programs suggest that short‐term career development curricula can provide valuable training without affecting time‐to‐degree.

It is clear that additional programs are needed to meet the changing demands of the biomedical workforce. In 2017 and 2019, Yale University faculty in the Combined Program for the Biological and Biomedical Sciences (BBS) offered a semester‐long course entitled “Skills Development for Diverse Scientific Careers” through funding provided by NIGMS as a supplement to training grant T32GM007223. This course was designed to intentionally address the national calls for broader skills‐based training for biological and biomedical scientists, while providing content not covered in any current curriculum at Yale. Here, we present the framework for the “Skills Development for Diverse Scientific Careers” course and evaluate trainee pre‐post course knowledge, interest, and confidence in obtaining a position within multiple nontraditional career trajectories.

## 2 MATERIALS AND METHODS

### 2.1 Program funding

This work was initiated in 2016 and supported by an Administrative Supplement to National Institutes of Health grant T32GM007223 (PD Susan Baserga), which served as an important catalyst to organize this course for the first time. Course evaluation was approved by the Yale Institutional Review Board (#2000024769 & #2000026711).

### 2.2 Participant recruitment

The course was offered as an optional elective that required registration (BBS 550b), and met once/week for 90 min throughout the spring semester. Course credit (Satisfactory/Unsatisfactory) was awarded to students who attended 80% of the sessions. This flexible approach allowed for student engagement with minimal additional time burden. The course was advertised via the Biological and Biomedical Sciences (BBS) newsletter, through the MD/PhD program, by each graduate department, and on posted fliers. Course materials were distributed via a web‐based system for each session, and the slides or resources were posted to a central website https://medicine.yale.edu/bbs/training/nihprograms/cmb/cmbcardevops/ for any BBS student to view with the permission of the presenter.

### 2.3 Course development

We ran the “Skills Development for Diverse Scientific Careers” course over 10 weeks during spring 2017 and 2019, meeting once/week at the end of the day for 90 min. Engaging speakers were chosen according to their expertise in the diverse content areas aligned to the most common career sectors of Yale University BBS graduate students using the Yale Institutional Training Grant Database's alumni section with data from the past 25 years (Table 1). To see the current percentages of graduate career sectors as recorded in the database, please see https://medicine.yale.edu/bbs/career/outcomes/. For the second course offering, additional career trajectories were added to expand the multiplicity of career paths. Each class period was designed to include approximately 45 min of presentation from the invited speaker and 45 min of active learning and discussion. Speakers were instructed to allow ample time for questions and interactions.

**TABLE 1:** How well did the “Skills Development for Diverse Scientific Careers” course cover common career sectors among biomedical PhD graduates?

	Career sector	Directly addressed in 2017 course offering (yes/no)	Directly addressed in 2019 course offering (yes/no)
1	Academic (research intensive)	Yes	Yes
2	Academic (teaching intensive)	No	No
3	Academic (other job type)	Yes	Yes
4	Business/entrepreneurship	Yes	Yes
5	Consulting	No	No
6	Law or finance	Yes	Yes
7	Government or non‐profit	No	No
8	Healthcare or clinical	Yes	Yes
9	K‐12 education	No	No
10	Library science	No	Yes
11	Pharmaceuticals or biotechnology	Yes	Yes
12	Publishing/communications	No	No

The “Skills Development for Diverse Scientific Careers” course was designed around four specific course objectives (Table 2). Upon completion, students would be able to think broadly about a range of career options and gain confidence to align their own career objectives with the skills needed for those fields. In developing weekly sessions, we examined the curriculum to design content that addressed career‐related topics not covered in any curriculum at Yale. In addition, we developed sessions to mirror the 12 most common career sectors of Yale University BBS graduate students using the Yale Institutional Training Grant Database's alumni section (Table 1). Topics included: how to run clinical trials both in academia and in pharma, the business and scientific sides of biotech, strategies for optimal professional productivity, how to convert a CV into a resume, resilience for early career scientists, and more (Supplemental Items 1 and 2 for both syllabi). The first course offering held many sessions surrounding careers in industry, as interest in this career path is prevalent among biomedical trainees.^25^ The second course offering expanded the number of career paths to include additional fields such as library science and biosafety (Table 1).

**TABLE 2:** Course objectives for “Skills Development for Diverse Scientific Careers” course

Course objectives	After completing this course, students should be able to
1	Understand nontraditional biomedical career opportunities in research‐related careers.
2	Think broadly about their career options and what skills are required across sectors.
3	Synthesize personal career interests with the information and resources available about various career sectors.
4	Identify next steps for their career path after graduation with greater confidence in the necessary skills for diverse positions.

### 2.4 Course evaluation and survey design

Course evaluation was completed using attendee surveys aligned with course objectives (Table 2). During the 2017 pilot course, five questions were administered to attendees through an email link after each class using an online survey tool. The questions were: “Did the presentation expose you to new options for your future career in biomedical science?” “Was it clear what roadmap such a career path might take?” “Are you considering this option as a potential career path?” “Was it clear how you would gain the necessary skills?” “Was there adequate time for discussion and interaction with the speaker?” These responses were used to build course objectives, and a robust evaluation plan for the 2019 course (Table 2, Supplemental Items 3, 4, 5).

Evaluations for the 2019 course offering were updated to utilize a pre‐post survey method and were developed by conducting a comprehensive literature review of current assessment instruments used to evaluate graduate student learning. Surveys were built using an online survey software (Qualtrics), with the pre‐survey administered the week before the class began. Students who had registered for the course received an email with the link to the survey, an explanation of the rationale for collecting their responses, and a questionnaire asking participants for consent to use their data (Supplemental Item 3). The post‐course survey link was emailed to all registered attendees after the final class of the semester, with reminders emailed out for 2 weeks after the class ended (Supplemental Item 5).

The 2019 surveys were designed to align with the course objectives (Table 2 and Supplemental Items 4 and 5 for both pre‐ and post‐course surveys). The pre‐course survey asked students a combination of open‐ended and close‐ended responses using a Likert scale of strongly disagree to strongly agree to the statements provided.^25^ The questions included: what they hoped to learn from the course, departmental exposure to different career opportunities, confidence levels in knowing what steps they need to take for their career and knowing what skills are needed for a variety of positions.^19^ The survey also asked them about interest, knowledge, and confidence in their ability to succeed in 12 career sectors, with an “other” option. The 12 categories were gleaned from the Yale Institutional Training Grant Database's alumni section of BBS graduate students who have reported the career sectors that they enter into upon graduation over the past 25 years (Table 1).

Students were also asked about perceived advisor and dissertation committee support for various career avenues, whom they have spoken with to discuss career goals, what barriers or challenges they have encountered when planning their next career steps, what resources they have used at Yale, and their sense of identity using a standardized scientific identity development scale.^13^, ^28^, ^29^ Finally, students answered five demographic questions about BBS track, year of study, gender, underrepresented group (UG) status, and citizenship.

The 2019 post‐course survey repeated questions about departmental exposure to different career opportunities, confidence levels in knowing what steps they need to take for their career and knowledge of the skills required in a variety of positions (Supplemental Item 5). The survey also included Likert questions about interest for, knowledge of the types of positions available, and confidence in ability to succeed in 12 career sectors, with an “other” option, as in the pre‐course survey.^25^ Students were again asked about perceived advisor and dissertation committee support for various career avenues, whom they have spoken with to discuss career goals, what resources they have used at Yale, and the scientific identity development scale for comparison with the pre‐course survey data. Additional questions asked students about what they learned during the course, whether the course helped them identify next steps in their career planning, and whether the course provided them with knowledge to guide their career decisions. Finally, we asked students which sessions were the most helpful for their career planning, what topics they wished they had learned more about, and what suggestions they have to improve the course in the future.

### 2.5 Data analysis

Qualitative and quantitative responses were analyzed to determine impact of the “Skills Development for Diverse Scientific Careers” course across multiple variables. Pre‐course and post‐course survey responses were linked using student's email addresses. All identifying information was removed prior to analysis. The open‐ended questions on both 2017 and 2019 surveys were thematically coded using NVIVO qualitative analysis software by the first authors.^30^ The responses were initially coded in relation to the original questions that were asked. Two norming and discussion sessions were held between the co‐first authors to develop a coding structure for both the 2017 additional comments, and the 2019 open‐ended questions. Within the responses for each question, grounded theory was utilized which enabled themes to emerge. Open coding resulted in several categories that were then refined further with axial coding. Due to the complexity of some of the student experiences, several of the responses were coded into multiple categories. Likert scale questions were compared using SPSS software (vs 24.0 for mac) for pre‐course and post‐course scores.

## 3 RESULTS

### 3.1 Sample

Sixty‐four students registered for the course across the two semesters it was offered. Table 3 depicts the summary demographics of the two semesters of students who registered for the course, and Table 4 portrays the departments that the students were affiliated with at the time of taking the course.

**TABLE 3:** Demographics of registered graduate students for the “Skills Development for Diverse Scientific Careers” course

	2017 (*n* = 35)	2019 (*n* = 29)	Total
Years in training			
First‐year	1	9	10
Second‐year	6	5	11
Third‐year	7	2	9
Fourth‐year	9	7	16
Fifth‐year	8	6	14
Sixth‐year	3	0	3
Seventh‐year	1	0	1
Gender			
Female	18	18	36
Male	17	11	28
Underrepresented Group status	17.14%	13.79%	10
Citizenship			
US citizen	22	13	
Permanent US resident	4	2	
Non‐US citizen	8	14	

**TABLE 4:** Departmental representation for the “Skills Development for Diverse Scientific Careers” course

	2017 (*n* = 35)	2019 (*n* = 29)	Total
Molecular Biophysics and Biochemistry	8	8	16
Molecular, Cellular and Developmental Biology	4	3	7
Immunology	2	4	6
Cell Biology	3	3	6
Experimental Pathology	3	2	5
Genetics	4	1	5
Interdepartmental Neuroscience Program	1	3	4
MD/PhD Program	3	0	3
Pharmacology	4	0	4
Computational Biology and Bioinformatics	1	2	3
Microbiology	1	0	1
Engineering and Applied Science	1	0	1

### 3.2 2017 Pilot data

**FIGURE 1:**
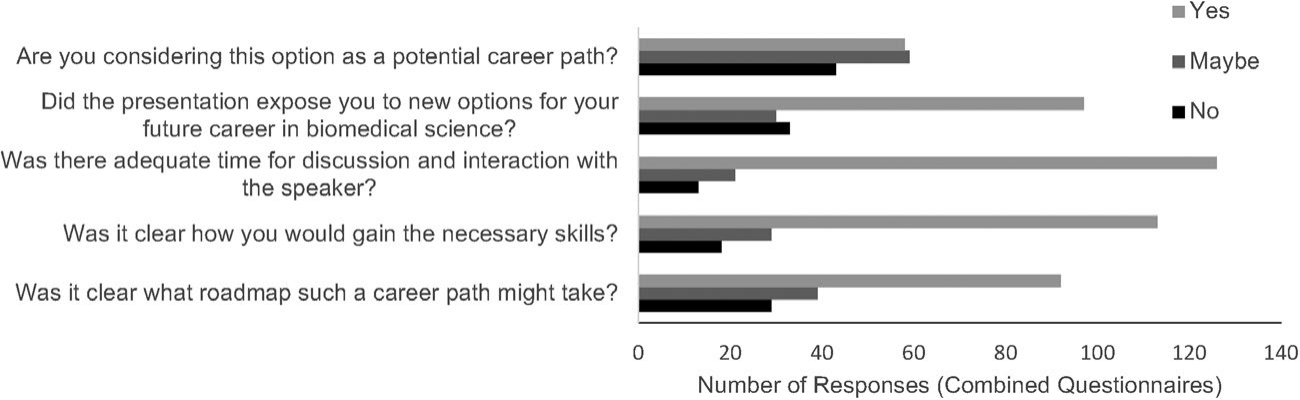
Summary of the responses to the “Skills Development for Diverse Scientific Careers” course in 2017. The number of yes (light gray), maybe (medium gray), and no (black) responses for the combined session questionnaires are indicated for each of the survey questions

**FIGURE 2:**
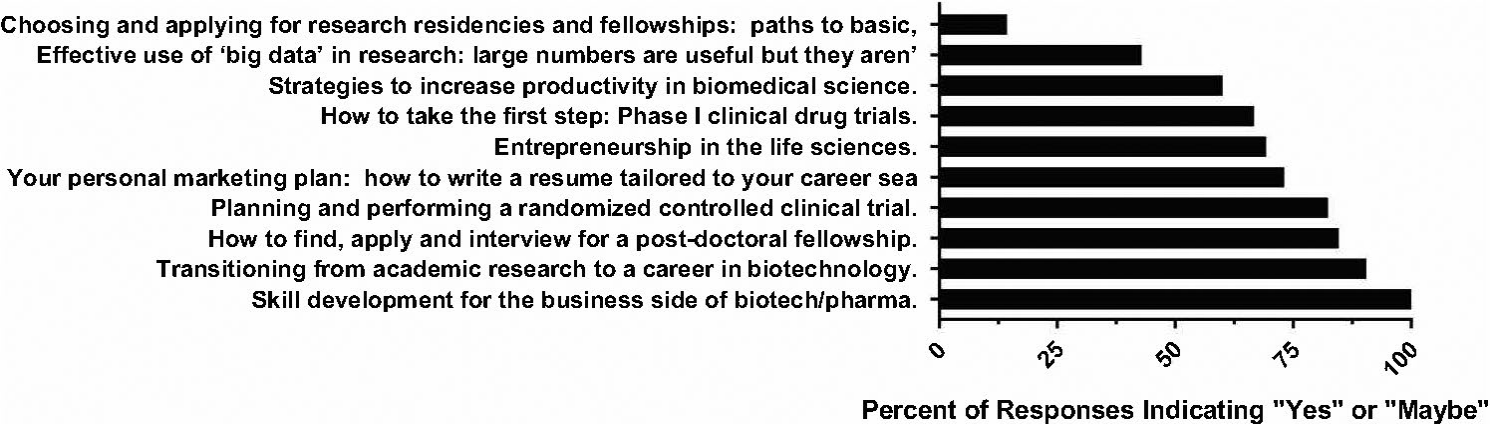
Percent interest in various career paths as reported on the 2017 “Skills Development for Diverse Scientific Careers” course surveys taken after each session

Preliminary data were collected after the first course offering in 2017. Participants were asked to fill out a five‐question survey at the end of each seminar they attended throughout the semester (Table 5 and Supplemental Item 1). Attendance for the seminars ranged from 31 (Transitioning from academic research to a career in biotechnology) to 19 (Entrepreneurship in the life sciences), with an average of 25 students per seminar. Response rates ranged from 92.86% (*n* = 26, how to write a resume) to 36.84% (*n* = 7, entrepreneurship), averaging 61.63%. Combining responses across all 10 seminars, 79.38% of attendees reported the presentation exposed them to new options for their future career in biomedical science (Figure 1). Interest in the topics varied with students responding “yes” or “maybe” at varying rates (Figure 2). Notably, over 50% of respondents were interested in 8 of the 10 seminars for their future careers, and 100% of respondents were interested in the business side of biotech/pharma as a potential career path (Figure 1).

**TABLE 5:** “Skills Development for Diverse Scientific Careers” course 2017 topics

Week	Topic
1	Transitioning from academic research to a career in biotechnology.
2	Your personal marketing plan: how to write a resume tailored to your career search.
3	Planning and performing a randomized controlled clinical trial.
4	Skill development for the business side of biotech/pharma.
5	Entrepreneurship in the life sciences.
6	Choosing and applying for research residencies and fellowships: paths to basic, translational and clinical research careers for physician‐scientists.
7	How to take the first step: Phase I clinical drug trials.
8	How to find, apply, and interview for a post‐doctoral fellowship.
9	Effective use of “big data” in research: large numbers are useful but they are not a cure all.
10	Strategies to increase productivity in biomedical science.

The chosen pedagogical approach of 45 min of presentation with 45 min of questions and interaction with the presenter was rated favorably by the students such that 91.88% (*n* = 147) participants reported there was adequate time for discussion and interaction with the speaker (Figure 1). In the post‐seminar surveys, 18.13% of participants reported that it was not clear what roadmap such a career path might take, with 11.25% of participants reporting it was not clear how they would gain the necessary skills for a career in that sector (Figure 1).

Students were asked an open‐ended question about additional comments from each seminar, and 55 comments were provided across the 10 seminars. Upon initial coding, the two raters determined four main themes and had 86% agreement^31^ across coded responses, with a Cohen's Kappa of *κ* = 0.72. The four themes that emerged were responses relevant to the content of the seminar (34.8%, *n* = 23), responses specific to the speakers (42.4%, *n* = 28), responses that demonstrated personal reflection (13.6%, *n* = 9) after the seminar ended, and suggestions (9.1%, *n* = 6) to improve the series. Upon discussion of the responses and codes, the authors were able to resolve many of the original discrepancies in coding and concluded the second discussion session coding with 94% agreement, *κ* = 0.87. Of these comments, 74.6% (*n* = 41) were positive, and 18.1% (*n* = 10) provided suggestions to improve areas of the course in either structure, such as more time for discussion, or additional content (selected quotes below):

This presentation was not about a career option, but instead on how to market our PhD's to various post‐graduate career options. I found the presentation well‐organized and extremely useful as I think about what I want to do and how I'm going to get there once I graduate. (Personal Reflection)

I thought this was a well‐designed lecture that clearly covered not only who should pursue a postdoctoral fellowship, but also steps that we need to take to acquire such a position. The presenter also tied in comments made by previous lecturers in this course, which I believe has helped me in thinking about and making the determination of whether a postdoctoral fellowship is the appropriate step for me to take for my career. (Speaker specific and Content related)

I felt that, while informative, the talk was catered to MD/PhDs (physician scientists) and was of little use for graduate students. What would have been more useful was if the presentation was focused on how basic scientists (most BBS PhDs) enter translational research as a future career path. (Suggestions for improving the series)

### 3.3 2019 Course evaluation data

Students (*n* = 12) that consented to share their data and filled out both the pre‐ and post‐course surveys are included in the following results, and reported the following demographic characteristics: 50% Female, 58.33% US citizen, 41.67% Asian, with 3.17 average years in training (range 1–5), and representation from five departments, including Molecular Biology and Biophysics, Interdepartmental Neuroscience Program, Molecular, Cell Biology, Genetics and Development, Computational Biology and Bioinformatics, and Experimental Pathology (Tables 3 and 4). The survey respondents were reflective of the course demographics, and the results are discussed below by course objective (Table 2). Across open‐ended questions and two discussion sessions between the first authors for coding of themes in the 2019 data, 98% agreement of themes was achieved, *κ* = 0.96. The high kappa is most likely attributed to the specific questions that were asked during this iteration of the course, with little variability in responses observed.

### 3.4 Objective 1: Understand nontraditional biomedical career opportunities in research‐related careers

On the 2019 pre‐course survey, 75% of students discussed wanting to obtain broader exposure to careers outside of academia (*n* = 9), in addition to learning new skills to land their next position (*n* = 5). Students were also asked what barriers or challenges they had encountered while planning their next career steps, and 50% of students (*n* = 6) reported they were unsure of which steps they should take, and 30% of students (*n* = 4) reported they needed more knowledge of available options outside of academia.

**FIGURE 3:**
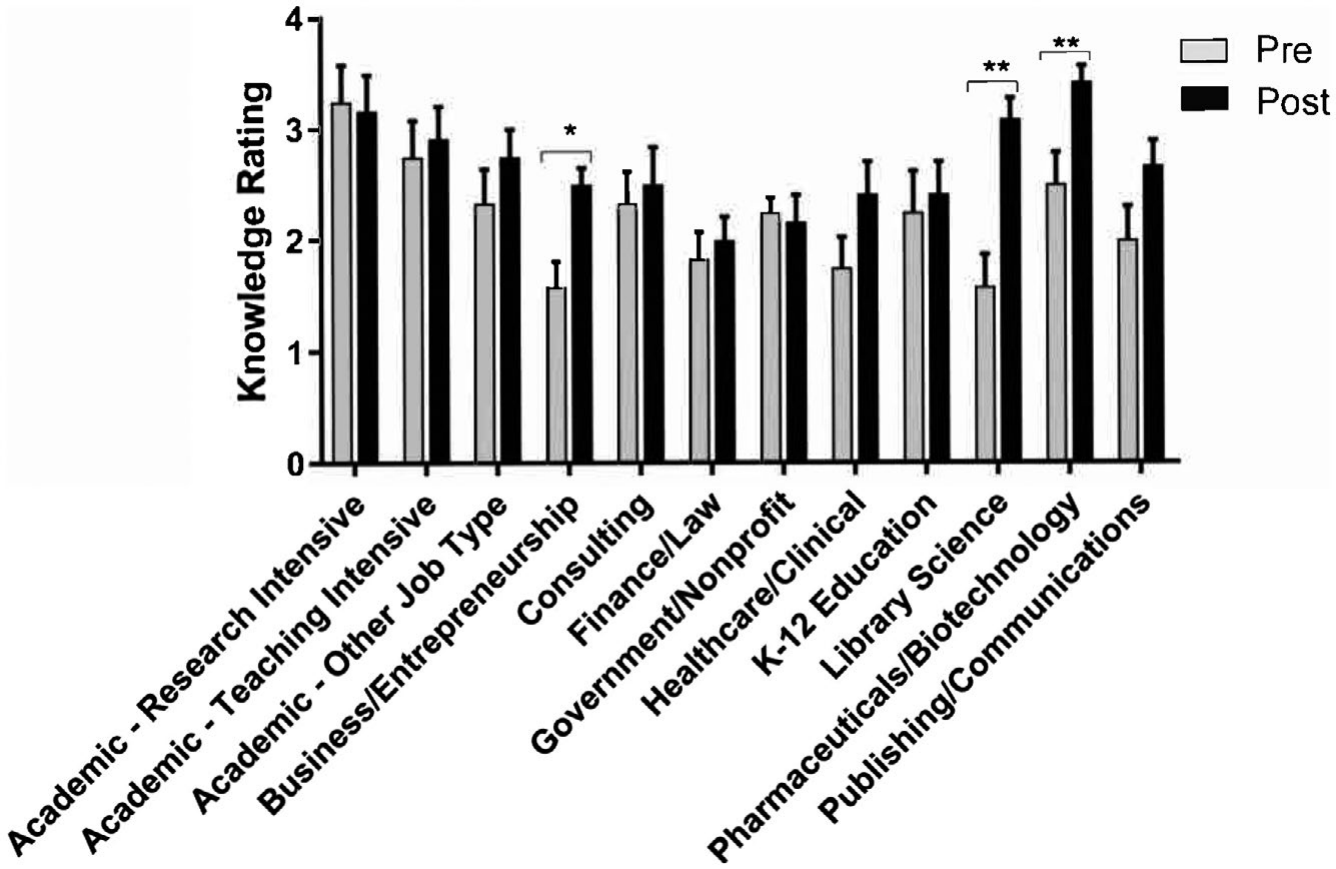
Reported knowledge ratings of multiple career sectors increased upon course completion. Pre‐course survey ratings are shown in light gray, while post‐course survey ratings are shown in black. Significance was assessed using a paired sample *t*‐test in GraphPad Prism, *p < 0.05, **p < 0.01

Student responses across the pre and post evaluations reflected increases in student knowledge of the variety of possible career avenues. Students rated their knowledge of available jobs across 12 alumni career sectors (Table 1). A paired samples *t* test was conducted to compare student's knowledge of the sectors before the course began to after it concluded, and four sectors were significantly different in knowledge ratings: Government or non‐Profit, *t*(9) = 3.28, *p* = 0.009; K‐12 Education, *t*(9) = 2.86, *p* = 0.019; Library Science, *t*(10) = 4.95, *p* = 0.001; and Publishing/Communications, *t*(10) = 2.283, *p* = 0.046 (see Figure 3). These data were further supported by individual comments from several respondents, including the selected comments below:

The most important part of this course for me was learning about the various careers that life science PhDs have gone into after graduate school.

This course has made me consider a variety of career options. Right now, I want to determine which career path would make me the happiest and start working towards becoming qualified for such a position.

Students were asked whether their department exposed them to a variety of career options, and a paired samples *t*‐test was conducted to compare students’ perceptions before and after the course. There was a significant increase in departmental exposure when comparing the start of the course (*M* = 2.42, SD = 0.79) to its conclusion (*M* = 3.17, SD = 0.58); *t*(11) = 3.00, *p* = 0.012. This result suggests that during the semester, students felt that they were exposed by their department to more of a variety of career options. This result could be due to greater awareness by the department in supporting broad career interests, as faculty knew this course was running, and perhaps sought to augment other career development discussions and materials. It could also be the case that as students' awareness and interest grew during the semester, they engaged in more frequent or open conversations with departmental colleagues about understanding career opportunities after training.

### 3.5 Objective 2: Think broadly about their career options and what skills are required across sectors

The “Skills Development for Diverse Scientific Careers” course significantly expanded knowledge about diverse career options, with 100% of students (*n* = 11) reporting that this course helped them identify their next steps in career planning and 100% of students (*n* = 12) reporting that this course provided them with knowledge to help guide their career decisions in the future.

**FIGURE 4:**
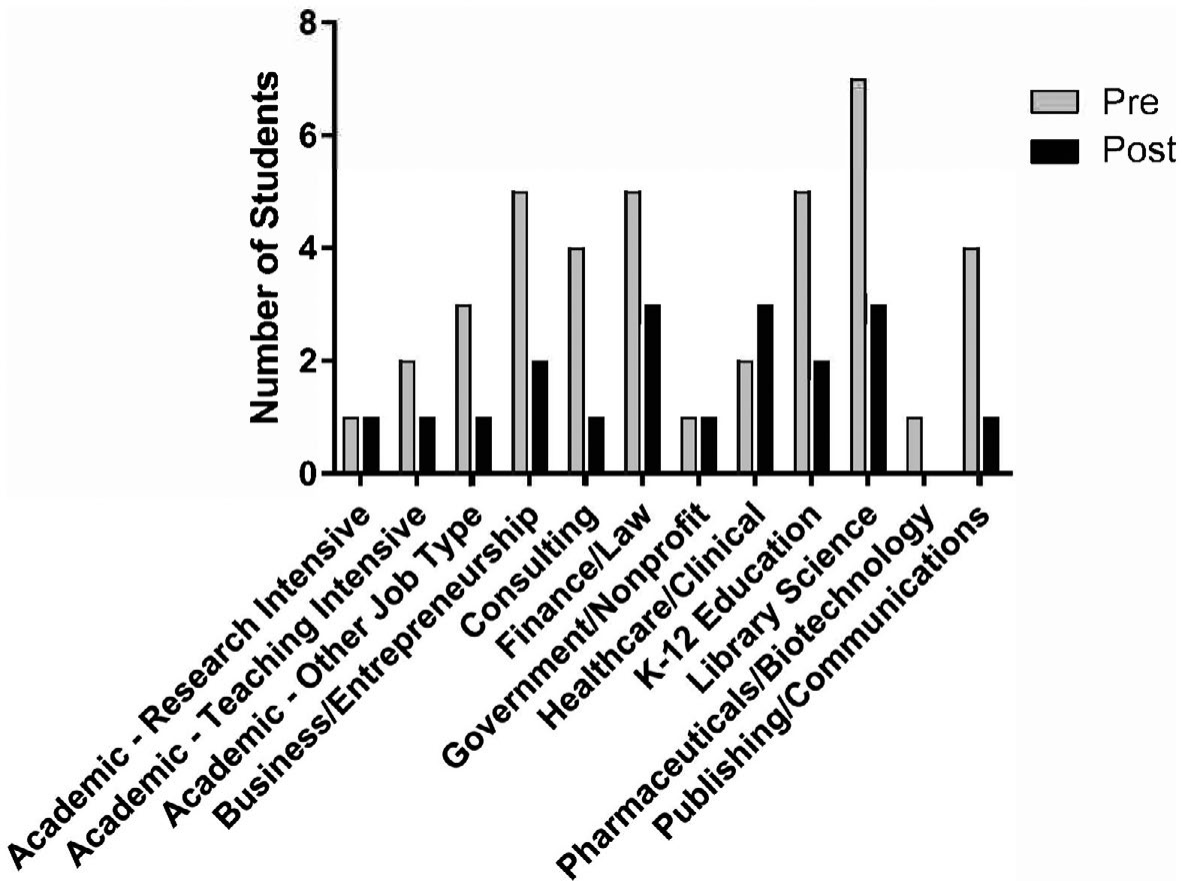
Graduate students’ interest in diverse careers increases with exposure to them (2019). The number of students reporting that they are “not interested” in the indicated career sectors decreased upon “Skills Development for Diverse Scientific Careers” course completion. The number of students “not interested” for each prospective field according to the pre‐course survey is shown in light gray, and the number of students indicating “not interested” in the post‐course survey is shown in black

Students reported their interest in a variety of career sectors, and of note was that over the course of the semester, students shifted their interest levels in 10 out of the 12 career sectors (Figure 4). In the pre‐survey, 27.78% of the career sector categories were designated by the students that they were “not interested”, however on the post‐course survey, this percent of reports of not being interested in career sectors decreased to 13.19%. For certain topics, such as the broader skills‐based sessions, the existing survey questions may have been unclear. For example, a student may have answered “no” for interest in the field for the resume writing session simply because the session did not focus on a specific career. However, the overall data demonstrate that the course resulted in increased knowledge of available careers while simultaneously enhancing interest in these new paths.

### 3.6 Objective 3: Synthesize personal career interests with the information and resources available about various career sectors

**FIGURE 5:**
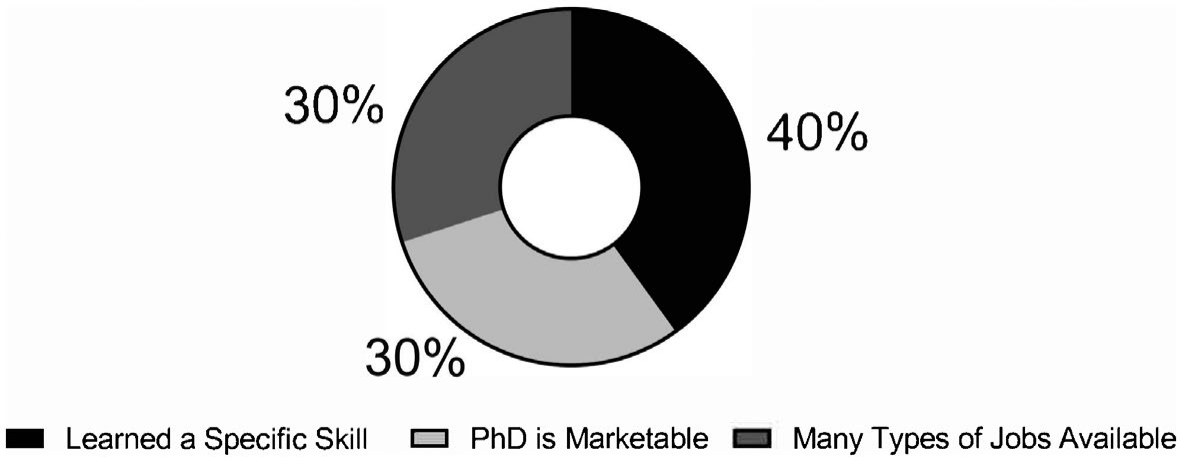
Student responses on the most important thing they learned from the “Skills Development for Diverse Scientific Careers” course (2019)

On the post survey, students reflected on the most important information they learned from the course (Figure 5). Of the respondents, 30% (*n* = 3) stated that they learned that their PhD is very marketable and that there are numerous available jobs in the biological and biomedical sciences. Additionally, 40% of respondents (*n* = 4) discussed that they learned a specific skill, such as building resilience, or learned about the resources available to them as they discover where their career passions lie. When asked on the post survey about how this course specifically influenced their career goals, 56% of students (*n* = 5) responded that they were able to narrow down their career path or confirm the path they are on. One student responded that the course boosted their confidence on the job market, while another student responded that the course helped them start to plan their next career development steps.

When asked on the post survey which sessions were the most helpful for their career planning, four students found the session on biotechnology entrepreneurship to be the most helpful for their career planning, two students cited the session on resume writing presented by the Office of Career Strategy at Yale, and two cited the sessions on industry (see selected quotes below).

The sessions with Margaret Kiss and Will McLean were the most helpful for insight into the transition from academic to industry jobs initially focused on bench work before transitions into more managerial roles.

Resume building was helpful on how to tailor your PhD experience to a resume, and the start‐up founder was also helpful.

I really appreciated learning about the different positions available. More than that though, I liked talking about the requirements going into different jobs.

Yet, five students responded that they would have liked to learn more about available positions in government, pharmacology, consulting, finance or law, science policy, or non‐profits. When asked about strategies for improving the course, three respondents suggested adding additional content including careers in law, journalism, and science communication.

### 3.7 Objective 4: Identify next steps for their career path after graduation with greater confidence in necessary skills for diverse positions

**FIGURE 6:**
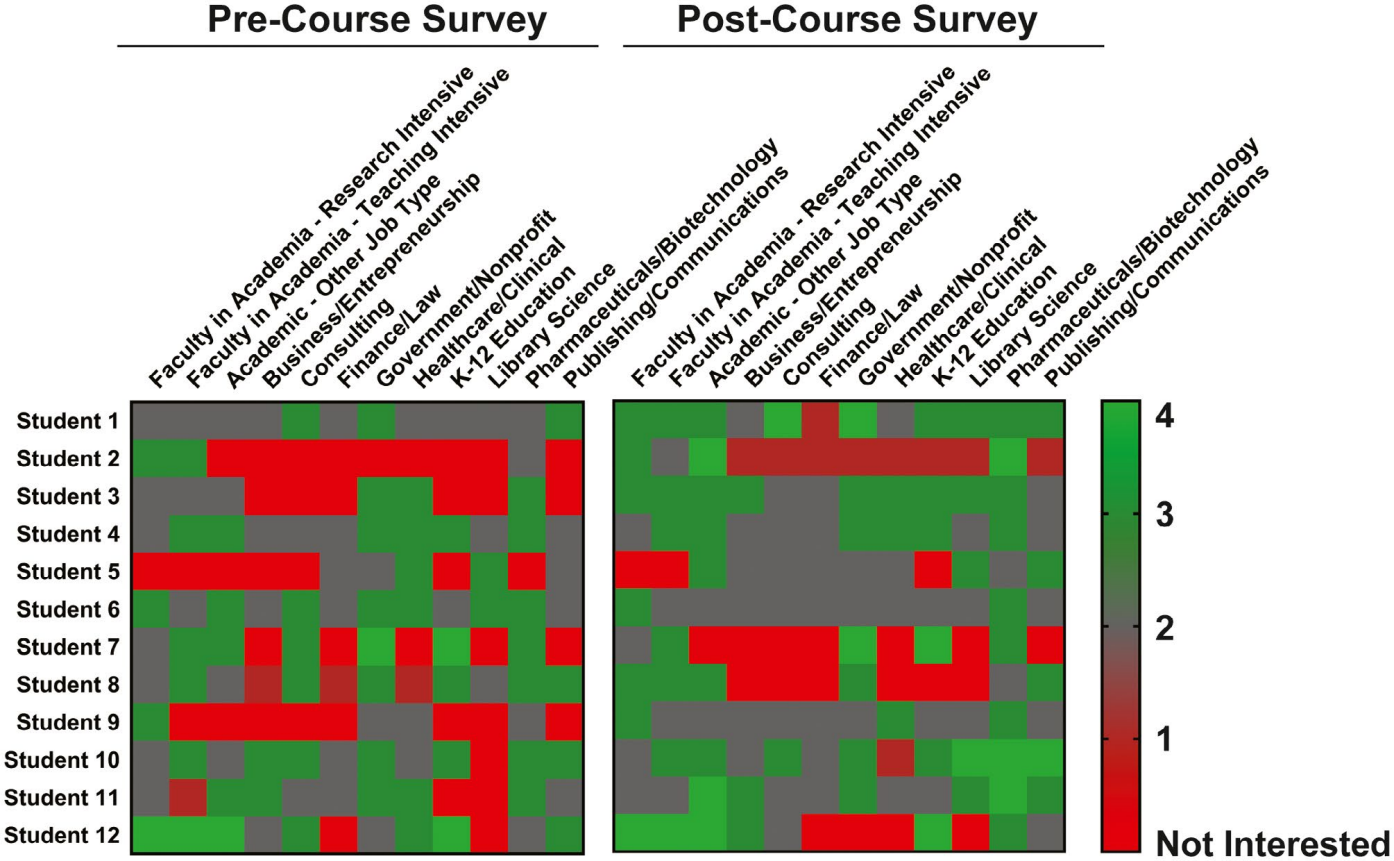
Student interest in their ability to succeed in 12 diverse career fields increases after completing the “Skills Development for Diverse Scientific Careers” course. A heatmap showing changes in student interest between the pre‐course and post‐course surveys. The students ranked their interest on a scale from Not Interested (red) to Very Interested (4, green). Responses from the pre‐course survey for each of the 12 career options are shown on the left, and responses from the post‐course survey are shown on the right. The image was generated using the GraphPad Prism software [Colour figure can be viewed at wileyonlinelibrary.com]

Student confidence greatly increased across a variety of career sectors, including those not directly addressed within the course structure (Figure 6). A paired samples *t* test revealed that there was a significant increase in trainee confidence levels when comparing the start of the course (*M* = 1.92, SD = 0.52) to its conclusion ((*M* = 2.50, SD = .52); *t*(11) = 3.02, *p* = 0.012). A paired samples *t* test was also conducted to compare students’ confidence levels for knowing what skills are needed to obtain a position in the research workforce before and after the course. There was a significant increase in the confidence levels for before the course began (*M* = 1.92, SD = 0.52) and after the course ((*M* = 2.91, SD = .52); *t*(11) = 5.75, *p* = 0.000). This result suggests that students were more confident in knowing what steps to take and what skills they would need to obtain a position across various career sectors after the course ended. However, four students suggested that this could be improved upon by having the speakers focus more directly on how they transferred the skills learned during their PhD into their nontraditional careers.

When examining the standardized scale on scientific identity^27^ there were no significant differences (*p* > 0.05) in students’ reported scientific identity scores from pre (*M* = 3.82) to post (*M* = 3.87) course. Questions asked were along a five point Likert scale for strongly disagree to strongly agree that they: have a strong sense of belonging to the community of scientists, derive great personal satisfaction from working on a team that is doing important research, have come to think of themselves as scientists, belong in the field of science, and the daily work of a scientist is appealing to them.

The higher education environment can strongly affect student confidence in pursuing careers outside of academia. Therefore, we measured faculty support to better understand the mentor‐mentee relationships that factor into student perceptions of diverse career trajectories. We chose to run this course once a week at the end of the day to reduce conflicts with other courses or progress in the laboratory and did not hear of any faculty resistance to this format or to the content delivered, similar to research being done in this important area.^32^ On the post‐course survey, 100% of students (*n* = 12) reported they agreed or strongly agreed that their faculty advisor is supportive of them exploring various career avenues. Additionally, 90.91% of respondents (*n* = 10) agreed or strongly agreed their thesis or dissertation committee is supportive of them exploring various career avenues.

**FIGURE 7:**
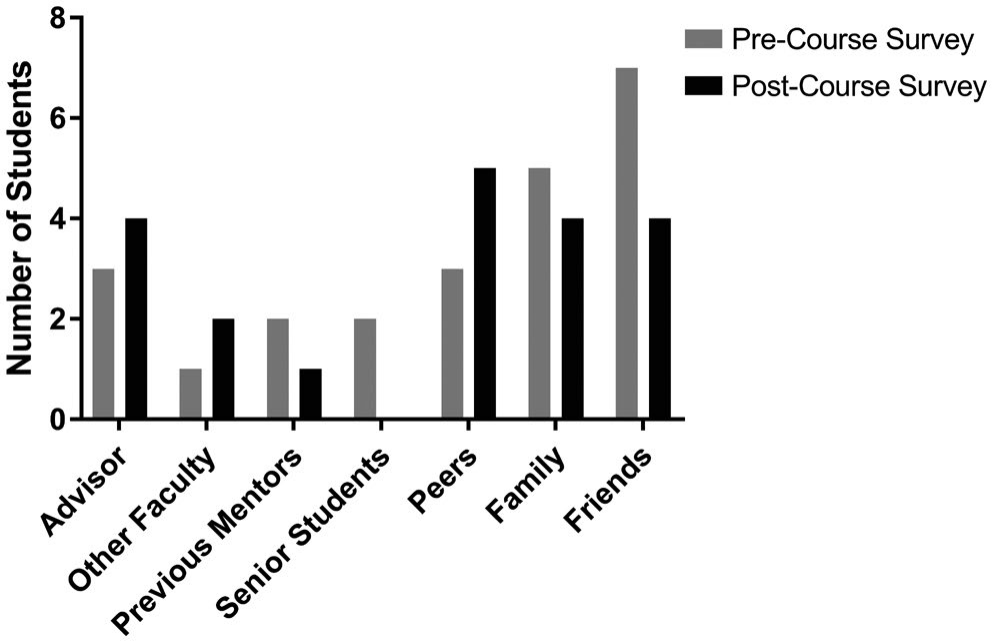
The number of graduate students reporting using varied support systems to discuss their career goals was not significantly altered from the pre‐“Skills Development for Diverse Scientific Careers” course (2019) survey (gray) to the post‐course survey (black). Image generated using GraphPad Prism software

Students were also asked who they have spoken with to discuss their career goals. The data suggest that participation in the “Skills Development for Diverse Scientific Careers” course had no significant effect on student outreach to others regarding career choices. Similar support structures were used prior to the course and after taking the course (Figure 7).

## 4 DISCUSSION

The “Skills Development for Diverse Scientific Careers” course successfully exposed students to a broad array of nontraditional careers in the biological and biomedical sciences. This one semester initiative held in the spring of 2017 and 2019 provided knowledge and skills‐based training that increased student's levels of confidence in navigating the next steps in their career trajectories after graduate school. The chosen pedagogical approach of 45 min of didactic lecturing, followed by 45 min of interactions and questions/answers with the presenter was well received by the students.

Students who attended the 2019 course offering reported significant increases in knowledge of four specific career sectors including government or non‐profit, K‐12 education, library science, and publishing or communications (Figure 3). There was also an increase in student interest for 10 out of 12 career sectors as a result of taking the course (Figure 4). Finally, student confidence was increased in terms of knowing what next steps to take and what skills are needed to obtain positions across the 12 career sectors (Figure 6). Notably, the surveys indicated changes in interest and confidence for topics not explicitly covered within the course sessions (Figures 3 and 4, Supplemental Items 4 and 5). This suggests that students learned skills and career self‐efficacy^33^ to allow them to broaden their fields of view beyond the course structure itself. We conclude that the course successfully met all four course objectives (Table 2) and helped to prepare graduates for careers in a variety of biomedical fields.

This course was conceived by Susan Baserga, co‐author on this paper, who saw a growing need for providing these skills and content to trainees, as she supervises the largest training program for graduate students at Yale in the sciences. Discussions with co‐creators, Anthony Koleske (then Director of the Biological and Biomedical Sciences), and Barbara Kazmierczak (Director of the MD/PhD Program), provided the foundation for developing this content as a one‐semester course. While the one‐semester course format worked well for this population of students, we believe that faculty at other institutions would be able to successfully implement these skills and concepts into other general graduate training courses. This approach would provide the content graduate students need to better equip them in navigating diverse careers, while reducing the logistics of implementing a full course. Our faculty did not have additional specialized training as they developed and executed this course, rather they brought in biological science PhDs who had taken different career paths to impart their knowledge to students.

It is interesting to note that the lack of finding evidence of difference in scientific identity from pre‐course to post‐course may demonstrate either the strength of identity current BBS students have in their programs, or present a limitation of this skills development course to nudge identity development in a meaningful way.^28^ The former would suggest that participants in this study feel that they belong in science, even if they do not have concrete career aspirations in a specific STEM area.

Elective courses, such as this one, allow students time and space to discover their vocation, help them to understand their skill sets, and give them the skills to address gaps in competency. Notably, offering a semester‐long elective course does not affect time‐to‐degree (5.8 years for course participants, *n* = 31; approximately 5.8 years for all students in the CMB training program at Yale). The course was run for only 90 min once per week at the end of the day,^25^ which may have led to increased student enrollment as it did not significantly burden students' time. Understanding that the timing of a course will never be ideal for all interested students, the ability to promote and deliver content to reach a broader audience at Yale is a priority in future offerings. The design of the “Skills Development for Diverse Scientific Careers” at Yale University provides an example of how institutions can build a semester‐long initiative to expose students to various career sectors and to reflect on the transferable skills that they may apply from their PhDs to positions outside of traditional academic routes.

Overall, the small sample is limited to students in the BBS and MD/PhD programs at Yale University, and survey responses were relatively low across both spring semesters. Logistically, the course can only be offered every other year at this time, and faculty noted that the first‐year students participating in 2019 were not able to engage well with the material. This suggests that, perhaps, first‐year students may need to focus on acclimating to graduate school and their academic journey prior to taking the course. In the future, we plan to restrict course participants to second‐year through fifth‐ or sixth‐year graduate students to best serve the students that may be better prepared to explore their own career goals and how their strengths relate to the career opportunities available.

In future offerings of the “Skills Development for Diverse Scientific Careers” course, we plan to add additional content that covers practical applications of ways to market skills learned during graduate school to various biomedical career paths. We also hope to add speakers from career paths not yet covered by the course, including law and science policy. We are currently exploring ways that this course could improve upon guiding students to use all available support structures. One way to do this may be to offer content describing how to conduct informational interviews, in the hopes that students will gather multiple viewpoints on their own, outside of the course, from a variety of sources. Future iterations of the course will take advantage of the MyIDP tool^34^, ^35^ to help students better reflect on how what they learned in the course correlates with their own strengths, weaknesses, and interests. We will also develop intentional assessments to better understand how students are integrating the content into their required MyIDP plans.

The one semester course in broad skills‐based training and knowledge of nontraditional career sectors for biological and biomedical scientists successfully ran twice at Yale University. Future iterations of the course will continue to push the boundaries on curricular offerings and invite prominent speakers from a variety of fields to present to students on the myriad opportunities available to them as a result of their training. Top priorities will be to continue querying students about the best ways to support them through their academic journey and to build accessible content that aligns with student needs. It is our hope that other institutions will use this course as a framework to expand student interest and confidence in a number of biomedical career paths worldwide.

## CONFLICT OF INTEREST

The authors declare no conflicts of interest.

## AUTHOR CONTRIBUTIONS

J. Claydon and SJ. Baserga designed and performed research. KI. Farley‐Barnes, J. Claydon, and SJ. Baserga analyzed data, wrote, and edited the manuscript.

## Supporting information

Supplementary Material

## References

[1] National Center for Science and Engineering Statistics (NCSES) . Doctorate Recipients from U.S. Universities: 2019. NSF 21‐308. National Science Foundation; 2020. https://ncses.nsf.gov/pubs/nsf21308/

[2] American Association of University Professors . Data snapshot: Contingent faculty in US Higher Ed; 2018. https://www.aaup.org/news/data‐snapshot‐contingent‐faculty‐us‐higher‐ed#.XZYfRy2ZOi5

[3] National Academies of Sciences, Engineering and Medicine . Graduate STEM Education for the 21st Century. The National Academies Press; 2018. doi:10.17226/25038

[4] Polka JK , Krukenberg KA , McDowell GS . A call for transparency in tracking student and postdoc career outcomes. Mol Biol Cell. 2015;26(8):1413‐1415. doi:10.1091/mbc.E14-10-1432 25870234 PMC4395122

[5] Larson RC , Ghaffarzadegan N , Xue Y . Too many PhD graduates or too few academic job openings: the basic reproductive number R_0_ in academia. Syst Res Behav Sci. 2014;31(6):745‐750. 25642132 10.1002/sres.2210 PMC4309283

[6] Golde CM , Dore TM . At Cross Purposes: What the Experiences of Doctoral Students Reveal About Doctoral Education. The Pew Charitable Trusts; 2001. www.phd‐survey.org/report%20final.pdf. Accessed June 20, 2011.

[7] Gibbs KD Jr , Griffin KA . What do I want to be with my PhD? The roles of personal values and structural dynamics in shaping the career interests of recent biomedical science PhD graduates. CBE—Life Sci Education. 2013;12:711‐723. 10.1187/cbe.13-02-0021 PMC3846521 24297297

[8] Leshner AI . Student‐centered, modernized graduate STEM education. Sci. 2018;360(6392):969‐970. doi:10.1126/science.aau0590 29853676

[9] Alberts B , Kirschner MW , Tilghman S , Varmus H . Rescuing US biomedical research from its systemic flaws. Proc Natl Acad Sci USA. 2014;111(16):5773‐5777. 24733905 10.1073/pnas.1404402111 PMC4000813

[10] Leshner AI . Rethinking graduate education. Sci Editorial. 2015;349(6249):349. doi:10.1126/science.aac9592 26206902

[11] Blume‐Kohout M . On What Basis? Seeking Effective Practices in Graduate STEM Education. National Academies of Sciences, Engineering and Medicine; 2007:1‐31.

[12] Denecke D , Feaster K , Stone K . Professional Development: Shaping Effective Programs for STEM Graduate Students. Council of Graduate Schools; 2017.

[13] Furhmann CN , Halme DG , O’Sullivan PS , Lindstaedt B . Improving graduate education to support a branching career pipeline: recommendations based on a survey of doctoral students in the basic biomedical sciences. CBE—Life Sci Education. 2011;10:239‐249. 10.1187/cbe.11-02-0013 PMC3164563 21885820

[14] Betz NE . Self‐efficacy theory as a basis for career assessment. J Career Assess. 2000;8(3):205‐222.

[15] de Valero YF . Departmental factors affecting time‐to‐degree and completion rates of doctoral students at one land‐grant research institution. J Higher Education. 2001;72(3):341‐367. doi:10.2307/2649335

[16] Lunsford L . Doctoral advising or mentoring? Effects on student outcomes. Mentor Tutoring: Partnersh Learning. 2012;20(2):251‐270. doi:10.1080/13611267.2012.678974

[17] Markowitz DG , DuPre MJ . Graduate experience in science education: the development of a science education course for biomedical science graduate students. CBE—Life Sci Education. 2007;6:233‐242. 10.1187/cbe.07-01-0004 PMC1964529 17785406

[18] Martinsuo M , Turkulainen V . Personal commitment, support and progress in doctoral studies. Stud High Education. 2011;36(1):103‐120. doi:10.1080/03075070903469598

[19] Sinche M , Layton RL , Brandt PD , et al. An evidence‐based evaluation of transferrable skills and job satisfaction for science PhDs. PLoS ONE. 2017;12(9):e0185023. doi:10.1371/journal.pone.0185023 28931079 PMC5607200

[20] Verderame MF , Freedman VH , Kozlowski LM , McCormack WT . Competency‐based assessment for the training of PhD students and early‐career scientists. eLife. 2018. doi:10.7554/eLife.34801.001 PMC6002247 29848440

[21] Gammie A , Gibbs K , Singh S . Early notice: new NIGMS institutional predoctoral training grant funding opportunity announcement – NIGMS feedback loop blog – National Institute of General Medical Sciences. 2017. https://loop.nigms.nih.gov/2017/03/early‐notice‐new‐nigms‐institutional‐predoctoraltraining‐grant‐funding‐opportunity‐announcement/

[22] National Institutes of Health . Biomedical research workforce working group report; 2012. https://acd.od.nih.gov/documents/reports/Biomedical_research_wgreport.pdf

[23] National Institute of General Medical Sciences . Ruth L. Kirschstein National Research Service Award (NRSA) Predoctoral Institutional Research Training Grant (T32). https://grants.nih.gov/grants/guide/pa‐files/PAR‐17‐341.html

[24] Meyers FJ , Mathur A , Fuhrmann CN , et al. The origin and implementation of the Broadening Experiences in Scientific Training programs: an NIH common fund initiative. FASEB J. 2016;30:507‐514. www.fasebj.org 26432783 10.1096/fj.15-276139 PMC6188226

[25] Lenzi RN , Korn SJ , Wallace M , Desmond NL , Labosky PA . The NIH "BEST" programs: institutional programs, the program evaluation, and early data. FASEB J. 2020;34(3):3570‐3582. doi:10.1096/fj.201902064 31960495

[26] Petrie KA , Carnahan RH , Brown AM , Gould KL . Providing experiential business and management training for biomedical research trainees. CBE—Life Sci Education. 2017;16:51. doi:10.1187/cbe.17-05-0074 PMC5589431 28798213

[27] Schnoes AM , Caliendo A , Morand J , et al. Internship experiences contribute to confident career decision making for doctoral students in the life sciences. CBE—Life Sci Education. 2017;17: doi:10.1187/cbe.17-08-0164 PMC6007763 29449270

[28] Anderson CB , Lee HY , Byars‐Winston A , Baldwin CD , Cameron C , Chang S . Assessment of scientific communication self‐efficacy, interest, and outcome expectations for career development in academic medicine. J Career Assess. 2016;24(1):182‐196. doi:10.1177/1069072714565780 26924920 PMC4764330

[29] St. Clair R , Hutto T , MacBeth C , Newstetter W , McCarty NA , Melkers J . The “new‐normal”: adapting doctoral trainee career preparation for broad career paths in science. PLoS ONE. 2017;12(5):e0177035. doi:10.1371/journal.pone.0177035 28542304 PMC5443479

[30] QSR International . NVivo Qualitative Data Analysis Software; 1999. Available from https://qsrinternational.com/nvivo/nvivo‐products/

[31] McHugh ML . Interrater reliability: the kappa statistic. Biochem Med. 2012;22(3):276‐282. PMC3900052 23092060

[32] Watts SW , Chatterjee D , Rojewski JW , et al. Faculty perceptions and knowledge of career development of trainees in biomedical science: what do we (think we) know? PLoS ONE. 2019;14(1):e0210189. 10.1371/journal.pone.0210189 30699144 PMC6353103

[33] Bullock‐Yowell E , Andrews L , Buzzetta ME . Explaining career decision‐making self‐efficacy: personality, cognitions, and cultural mistrust. Career Development Q. 2011;59:400‐411.

[34] Hobin JA , Fuhrmann CN , Lindstaedt B , Clifford PS . You need a Game Plan. 2012. Available from https://www.sciencemag.org/careers/2012/09/you‐need‐game‐plan

[35] Furhmann CN , Hobin JA , Lindstaedt B , Clifford PS . American Association for the Advancement of Science. 2021. Available from Myidp.sciencecareers.org

