# Supplementary material for: Building skill‐sets, confidence, and interest for diverse scientific careers in the biological and biomedical sciences
Source: FASEB Bioadv. 2021 Sep 15;3(12):998–1010. doi: 10.1096/fba.2021-00087 (PMC8664047; doi:10.1096/fba.2021-00087)
Supplement: Supplementary file 1 — Supplementary Material [file FBA2-3-998-s001.pdf]

Supplemental Items

Supplemental Item 1. 2017 Skills Development for Diverse Scientific Careers syllabus.

New Course in the Biological and Biomedical Sciences Skill Development for Diverse Scientific Careers— Spring 2017 B&BS 550b

Instructors: Faculty from Yale and the local biotechnology and pharmaceutical community Organizer: Susan Baserga (in charge); Anthony Koleske and Barbara Kazmierczak, co-organizers
Sponsored by: Administrative supplement to T32GM007223 and the BBS. Location: January 17 – May 2, 2017; SHM C103 (333 Cedar Street)
Time: Tuesdays, 5:00 to 6:30 PM

In the Spring of 2017 we will offer a new course that will address topics that are not currently covered in any curriculum at Yale: biotechnology entrepreneurship; how to run clinical trials, both in academia and in pharma; the business and scientific sides of biotech; strategies for optimal professional productivity; how to convert a CV into a resume; and how to find a post-doctoral fellowship or research residency.

Course Objectives: After completing this course, students should be able to:

1. Understand non-traditional biomedical career opportunities in research-related careers.
2. Think broadly about their career options and what skills are required across sectors.
3. Reflect on their MyIDP with individual strengths and weaknesses related to various biomedical research-related career options.
4. Synthesize personal career interests with the information and resources available about various career sectors.
5. Identify next steps for their career path after graduation with greater confidence in the necessary skills for diverse options in the biomedical workforce.

January 17, 2017 **Transitioning from academic research to a career in biotechnology.**
Instructor: Margaret Kiss, Ph.D., Director of Molecular Sciences, AxioMx, Inc.

January 24, 2017 **Your personal marketing plan: how to write a resume tailored to your career search.**
Instructor: Hyun Ja Shin, Ph.D., Office of Career Strategy.

January 31, 2017 **Planning and performing a randomized controlled clinical trial.**
Instructor: Loren Laine, M.D., Professor of Medicine (Gastroenterology).

February 7, 2017 **How to take the first step: Phase I clinical drug trials.**
Instructor: Patricia LoRusso, D.O., Professor of Medicine (Oncology).

February 28, 2017	**Skill development for the business side of biotech/pharma.**<br>*Instructor: Rachael Felberbaum, Ph.D., Senior Director of Business Development, Protein Sciences Corporation.*
March 28, 2017	**Entrepreneurship in the life sciences.**<br>*Instructor: Leonard Bell, M.D., Chairman of the Board of Directors, Alexion Pharmaceuticals, Inc.*
April 4, 2017	**Choosing and applying for research residencies and fellowships: paths to basic, translational and clinical research careers for physician-scientists.**<br>*Instructor: Barbara Kazmierzcak, M.D., Ph.D., Associate Professor of Medicine & Microbial Pathogenesis and the Director of the MD/PhD program.*
April 18, 2017	**How to find, apply and interview for a post-doctoral fellowship.**<br>*Instructor: Susan Baserga, M.D., Ph.D., Professor of Molecular Biophysics & Biochemistry, Genetics and Therapeutic Radiology; Program Director for the Predoctoral Program in Cellular and Molecular Biology.*
April 25, 2017	**Effective use of 'big data' in research: large numbers are useful but they aren't a cure all.**<br>*Instructor: Amy Justice, M.D., Ph.D., Professor of Medicine and of Public Health.*
May 2, 2017	**Strategies to increase productivity in biomedical science.**<br>*Instructor: Anthony Koleske, Ph.D., Professor of Molecular Biophysics and Biochemistry and Director of the Combined PhD Programs in the BBS.*

Supplemental Item 2. 2019 Skills Development for Diverse Scientific Careers syllabus.

Biological and Biomedical Sciences
Skill Development for Diverse Scientific Careers— Spring 2019
B&BS 550b

Instructors:	Faculty and staff from Yale and the local biotechnology and pharmaceutical community
Organizer:	Susan Baserga* (in charge); Anthony Koleske # and Barbara Kazmierczak, co-organizers. * Program Director # Trainer
Sponsored by:	Administrative supplement to T32GM007223 and the BBS.
Location:	January 15 – April 23, 2019; SHM C103 (333 Cedar Street)
Time:	Tuesdays, 5:00 to 6:30 PM

The Spring of 2019 is the second offering of a new course that will address topics that are not currently covered in any curriculum at Yale: biotechnology entrepreneurship; how to run clinical trials, both in academia and in pharma; the business and scientific sides of biotech; strategies for optimal professional productivity; how to convert a CV into a resume; and resilience for early career scientists. The course will be graded satisfactory/unsatisfactory based on 8/11 attendance. For feedback, please take the survey at the end of each session or speak with one of the organizers.

Course Objectives: After completing this course, students should be able to:

6. Understand non-traditional biomedical career opportunities in research-related careers.
7. Think broadly about their career options and what skills are required across sectors.
8. Reflect on their MyIDP with individual strengths and weaknesses related to various biomedical research-related career options.
9. Synthesize personal career interests with the information and resources available about various career sectors.
10. Identify next steps for their career path after graduation with greater confidence in the necessary skills for diverse options in the biomedical workforce.

January 15, 2019 — **Transitioning from academic research to a career in biotechnology.**
Instructor: Margaret Kiss, PhD, Director, Molecular Sciences, Abcam, Inc.

January 22, 2019 — **Careers in library science for PhDs.**
Instructor: Rolando Garcia-Milian, MLS, AHIP, Biomedical Sciences Research Support,
Lecturer in EPH.

January 29, 2019 — **Biosafety careers for PhDs.**
Instructor: Maren Schniederberend, PhD, Safety Advisor, Yale Environmental Health & Safety.

February 5, 2019 — **Strategies to increase productivity in biomedical science.**
Instructor: Anthony Koleske, Ph.D. #, Professor of Molecular Biophysics and Biochemistry and Director of the Combined PhD Programs in the BBS. #Trainer

February 12, 2019 **Preparing for the business side of biotech/pharma.**
Instructor: Rachael Felberbaum, PhD, Director, Sanofi Pasteur Transactions, Global Business Development & Licensing

February 19, 2019 **Effective use of ‘big data’ in research: large numbers are useful but they aren’t a cure all.**
Instructor: Amy Justice, M.D., Ph.D., Professor of Medicine and of Public Health.

February 26, 2019 No class

March 5, 2019 No class

March 26, 2019 **The practice of resilience for early career scientists.**
Instructor: Susan J. Baserga, MD, PhD, Professor, Molecular Biophysics & Biochemistry, Genetics, Ther. Rad.; Program Director, Predoctoral Program in Cellular and Molecular Biology, Yale University. *Program Director*

April 2, 2019 **Biotech start-up entrepreneurship**
Instructor: Will McLean, PhD, Co-founder and Vice President, Biology & Regenerative Medicine at Frequency Therapeutics, Inc.; Adjunct Assistant Professor in the Department of Surgery at the University of Connecticut School of Medicine.

April 9, 2019 **How to take the first step: Phase I clinical drug trials.**
Instructor: Patricia LoRusso, D.O., Professor of Medicine (Oncology).

April 16, 2019 **Your personal marketing plan: how to write a resume tailored to your career search.**
Instructor: Hyun Ja Shin, PhD, Office of Career Strategy.

April 23, 2019 **Grant funding for biotech-academia collaborations**
Instructor: Michel Ledizet, PhD, Senior Research Scientist, L2 Diagnostics.

Supplemental Item 3. Instructions and consent email for 2019 course surveys.

Instructions to Participants

Welcome to our survey! Thank you for taking the time to participate in this survey on the Skills Development Course for Diverse Scientific Careers. This survey was developed to help us evaluate this newly developed course, and to better understand how to support the diverse career interests of our graduate students.

This survey will take about 15 minutes to complete. You will not be compensated for participation. Responses are confidential. Your name, and email address will be collected to link your pre-course survey with your post-course survey data. You will receive an email reminder during the final week of this course to take the post-course survey. We appreciate your participation in both this pre-course survey and the post-course survey. After the data have been combined, all identifying information including your name and email address will be removed before any data analysis takes place. Your name and email address will not be reported at any level of survey analysis.

Your decision to participate, not answer questions or withdraw entirely from participation will not affect your academic standing, your relationship with your professors or future employability.

If you have any questions, please contact either Dr. Jennifer Claydon, Assistant Director of Training Program Assessment for the Biological and Biomedical Sciences and the Poorvu Center for Teaching and Learning, at Jennifer.claydon@yale.edu, or Dr. Susan Baserga, Principle Investigator at Susan.Baserga@yale.edu.

If you would like to talk with someone other than the researchers to discuss problems, concerns, or your rights as a research participant, you may contact the Yale University Human Subjects Committee, Box 208010, New Haven, CT 06520-8010, (203) 785-4688, human.subjects@yale.edu. Additional information is available at https://your.yale.edu/research-support/human-research/research-participants.

I have read and understand the information above and am older than 18 years of age. As such, I:

() Agree to participate
() Do not wish to participate

Supplemental Item 4. Career Survey Pre-Test questionnaire (2019).

Career Survey Pre-Test
Skills Development Course SP19

1. What do you hope to learn from taking this course?
2. Please rate your agreement with the following statements:
   My department has exposed me to a variety of different career opportunities.
   strongly disagree
   disagree
   agree
   strongly agree

3. I feel confident that I know the next step to take for my career.
   strongly disagree
   disagree
   agree
   strongly agree

4. I feel confident that I know the skills that are needed to obtain a position in the career field that is of interest to me.
   strongly disagree
   disagree
   agree
   strongly agree

5. Out of the following career fields, rate each field according to the statement:
   I am interested in pursuing a career in this field.

Type of Career	Strongly Disagree	Disagree	Agree	Strongly Disagree	Select your top two choices only
Faculty in academia at research intensive institution					
Faculty in academia at teaching intensive					
Academic other job type					
Business/Entrepreneurship					
Consulting					
Finance or Law					
Government or Non-Profit					
Healthcare or Clinical					
K-12 Education					
Library Science					
Pharmaceuticals or Biotechnology					

Publishing/Communications					
Other					

6. Out of the following career fields, rate each field according to the statement: I know what positions are available within this field.

Type of Career	Strongly Disagree	Disagree	Agree	Strongly Disagree
Faculty in academia at research intensive institution				
Faculty in academia at teaching intensive				
Academic other job type				
Business/Entrepreneurship				
Consulting				
Finance or Law				
Government or Non-Profit				
Healthcare or Clinical				
K-12 Education				
Library Science				
Pharmaceuticals or Biotechnology				
Publishing/Communications				
Other				

7. Out of the following career fields, rate each field according to the statement: I have confidence in my ability to succeed in a career in this field.

Type of Career	Strongly Disagree	Disagree	Agree	Strongly Disagree	Not Applicable
Faculty in academia at research intensive institution					
Faculty in academia at teaching intensive					
Academic other job type					
Business/Entrepreneurship					
Consulting					
Finance or Law					
Government or Non-Profit					
Healthcare or Clinical					
K-12 Education					
Library Science					
Pharmaceuticals or Biotechnology					
Publishing/Communications					
Other					

8. My faculty advisor is supportive of me exploring various career avenues.
   strongly disagree
   disagree
   agree
   strongly agree

9. My dissertation committee is supportive of me exploring various career avenues.
   strongly disagree
   disagree
   agree
   strongly agree

10. Who have you spoken with to discuss your goals for your career?

11. What barriers or challenges have you encountered while planning for your next career steps?

12. What other resources at Yale have you used as you continue to develop your career interests and goals?

Rate the degree you agree or disagree with the following statements concerning your sense of yourself as a scientist who undertakes research activities

	Strongly Disagree	Disagree	Neither Agree nor Disagree	Agree	Strongly Agree
I have a strong sense of belonging to the community of scientists	○	○	○	○	○
I derive great personal satisfaction from working on a team that is doing important research	○	○	○	○	○
I have come to think of myself as a 'scientist'	○	○	○	○	○
I feel like I belong in the field of science	○	○	○	○	○
The daily work of a scientist is appealing to me	○	○	○	○	○

Demographics:

1. What year are you in your graduate program?
2. What BBS track are you in?
3. Please select your gender:
   Male
   Female
   Transgender

Intergender
Prefer not to answer

4. Please indicate: are you Hispanic or Latino?
   Yes
   No
   Prefer not to answer

5. Please select one or more of the following races:
   American Indian or Alaska Native
   Asian
   Black or African American
   Native Hawaiian or Other Pacific Islander
   White
   Prefer not to answer

6. Please indicate your current status:
   US Citizen
   Permanent US Resident
   Non-US Citizen

Supplemental Item 5. 2019 career Survey Post-Test questionnaire.
Career Survey Post-Test
Skills Development Course SP19

1. My department has exposed me to a variety of different career opportunities.
   strongly disagree
   disagree
   agree Mell
   strongly agree

2. I feel confident that I know the next step to take for my career.
   strongly disagree
   disagree
   agree
   strongly agree

3. I feel confident that I know the skills that are needed to obtain a position in the research workforce that is of interest to me.
   strongly disagree
   disagree
   agree
   strongly agree

4. Out of the following career fields, rate each field according to the statement:
   I am interested in pursuing a career in this field.

Type of Career	Strongly Disagree	Disagree	Agree	Strongly Disagree	Select your top two choices only
Faculty in academia at research intensive institution					
Faculty in academia at teaching intensive					
Academic other job type					
Business/Entrepreneurship					
Consulting					
Finance or Law					
Government or Non-Profit					
Healthcare or Clinical					
K-12 Education					
Library Science					
Pharmaceuticals or Biotechnology					
Publishing/Communications					
Other					

5. Out of the following career fields, rate each field according to the statement:

I know what positions are available within this field.

Type of Career	Strongly Disagree	Disagree	Agree	Strongly Disagree
Faculty in academia at research intensive institution				
Faculty in academia at teaching intensive				
Academic other job type				
Business/Entrepreneurship				
Consulting				
Finance or Law				
Government or Non-Profit				
Healthcare or Clinical				
K-12 Education				
Library Science				
Pharmaceuticals or Biotechnology				
Publishing/Communications				
Other				

6. Out of the following career fields, rate each field according to the statement:
   I have confidence in my ability to succeed in a career in this field.

Type of Career	Strongly Disagree	Disagree	Agree	Strongly Disagree	Not Applicable
Faculty in academia at research intensive institution					
Faculty in academia at teaching intensive					
Academic other job type					
Business/Entrepreneurship					
Consulting					
Finance or Law					
Government or Non-Profit					
Healthcare or Clinical					
K-12 Education					
Library Science					
Pharmaceuticals or Biotechnology					
Publishing/Communications					
Other					

7. My faculty advisor is supportive of me exploring various career avenues.
   strongly disagree

disagree
agree
strongly agree

8. My dissertation committee is supportive of me exploring various career avenues.
   strongly disagree
   disagree
   agree
   strongly agree

9. Who have you spoken with to discuss your goals for your career?
10. What other resources or offices at Yale have you used as you continue to develop your career interests and goals?

11. What was the most important information that you learned during this course?

12. This course helped me identify next steps in my career planning.
    strongly disagree
    disagree
    agree
    strongly agree

13. This course provided me with knowledge that will help guide my career decisions in the future.
    strongly disagree
    disagree
    agree
    strongly agree

14. How has this course influenced your career interests?

15. What session of the course did you find most helpful to your career planning? Why was this session helpful for you?

16. What topics or career fields would you have liked to learn more about this semester?

17. What suggestions or changes do you have to improve this course?

Rate the degree you agree or disagree with the following statements concerning your sense of yourself as a scientist who undertakes research activities

	Strongly Disagree	Disagree	Neither Agree nor Disagree	Agree	Strongly Agree
I have a strong sense of belonging to the community of scientists	○	○	○	○	○
I derive great personal satisfaction from working on a team that is doing important research	○	○	○	○	○
I have come to think of myself as a 'scientist'	○	○	○	○	○
I feel like I belong in the field of science	○	○	○	○	○
The daily work of a scientist is appealing to me	○	○	○	○	○
